# SLC1A4 Promotes Malignant Transformation of Hepatocellular Carcinoma by Activating the AKT Signaling

**DOI:** 10.1155/ancp/1115184

**Published:** 2025-01-23

**Authors:** Jiaoyun Zheng, Jian Gong

**Affiliations:** ^1^Department of Pathology, The Second Xiangya Hospital, Central South University, Changsha 410011, Hunan, China; ^2^Department of Gastroenterology, The Second Xiangya Hospital, Central South University, Changsha 410011, Hunan, China; ^3^Research Center of Digestive Disease, Central South University, Changsha 410011, Hunan, China; ^4^Clinical Research Center of Digestive Diseases of Hunan Province, Changsha 410011, Hunan, China

## Abstract

Due to the difficulty in early diagnosis and the lack of treatment for advanced disease, the mortality rate of hepatocellular carcinoma (HCC) is high, and the 5-year overall survival rate is low at present. SLC1A4 is a neutral amino acid transporter, but its regulatory role and mechanism in HCC are still unclear. Through analyzing the TCGA database and clinical tissue specimens, this study uncovered the high expression of SLC1A4 in tumor tissues of HCC. Worse more, a high level of SLC1A4 may lead to a poor prognosis of HCC. Mechanically, silencing SLC1A4 inhibited the phosphorylation activation of AKT by suppressing the ubiquitin modification of AKT at lysine 63 and amino acid influx represented by D-serine, decreasing the protein level of *β*-catenin in the cell nucleus and suppressing the transcriptional activity of c-Myc and EpCAM promoters. As a result, silencing SLC1A4 inhibited the proliferation, migration, and stemness of hepatic cancer cells, which was successfully reversed by the introduction of exogenous AKT. Moreover, epithelial–mesenchymal transition (EMT) in vitro and metastasis potential in vivo of hepatic cancer cells was suppressed by the downregulated SLC1A4 level. In conclusion, SLC1A4 promotes the malignant transformation of HCC through activating signal transduction mediated by AKT. The findings in this study suggested that SLC1A4 may be a diagnostic indicator for the early HCC and a therapeutic target for the advanced HCC.

## 1. Introduction

As the sixth most common cancer and the third mortality of cancer-related death, liver cancer seriously endangers human health worldwide [1, 2], and 75%–85% of liver cancer are diagnosed as hepatocellular carcinoma (HCC) [3]. The main reason for the high mortality of HCC is the difficulty in early diagnosis and the lack of therapeutic methods for advanced disease [4–6]. For these reasons, the 5-year overall survival rate of HCC is only 18% worldwide, which is even worse in developing countries [7]. Exploring the pathogenesis of HCC is of great significance for improving the diagnostic efficacy of early HCC and the therapeutic efficacy of advanced HCC.

SLC1A4 (also named ASCT1) is a neutral amino acid transporter, participating in the transportations of alanine, serine, cysteine, threonine, glutamine, and so on [8, 9]. Through regulating the pathophysiological process of mitral valve prolapse and multiple system atrophy, SLC1A4 has been identified to be a potential therapeutic target [10, 11]. Meanwhile, mutations of SLC1A4 are associated with developmental delay, microcephaly, and hypomyelination [12, 13]. As for malignant tumors, the differentially expressed SLC1A4 has been identified to be a prognostic factor in breast cancer [14]. SLC1A4 and SLC1A5 transporters maintain glutamine absorption and utilization, contributing to cell proliferation driven by androgen receptors in prostate cancer [15, 16]. Through conducting Ubiscan quantification analysis in HepG2.2.15 cells, SLC1A4 has been found to reduce the levels of secreted HBsAg and HBeAg in cell culture supernatants [17]. Along with MAPK9, PCK2, ACSL3, STMN1, CDO1, and CXCL2, SLC1A4 is potentially associated with multiple oncogenic signatures and invasive-associated signaling pathways in HCC [18].

The analyses of integrative bioinformatics and functional profiling indicated that SLC1A4 may involve in regulating cancer phenotypes, immune regulation, and drug resistance in HCC [19], but the molecular mechanism regulated and oncogenic activity influenced by SLC1A4 in HCC still needs to be further explored. Through conducting in vitro and in vivo experiments, this study mainly revealed the oncogenic mechanism of SLC1A4 in HCC, illustrating the promoting role of SLC1A4 on the malignant transformation of hepatic cancer cells. The findings in this study may provide new sights for improving the diagnostic efficacy of early HCC and the therapeutic efficacy of advanced HCC.

## 2. Materials and Methods

### 2.1. Analysis of TCGA Database

Expression profiles of SLC1A4 in tumor tissues and adjacent tissues of HCC were analyzed based on TCGA database on website GEPIA (http://gepia.cancer-pku.cn/index.html) [20], and influence of SLC1A4 on 5-year overall survival of HCC was analyzed based on TCGA database on website Kaplan–Meier Plotter (https://kmplot.com/analysis/) [21].

### 2.2. Clinical Tissue Specimens

A total of 48 postoperative tissue specimens of HCC patients were collected for this study from January 2021 to December 2021 at The Second Xiangya Hospital. Prior to the surgery operation in which samples were collected, HCC patients had not received surgery, chemotherapy, radiotherapy, and immunotherapy. Protocols used for studying clinical tissue specimens were reviewed and approved by the Ethics Committee of The Second Xiangya Hospital, and written informed consent was provided by participated patients.

### 2.3. Cell Culture

The human hepatic cancer cell line Huh7 (RRID: CVCL_0336) was obtained from JCRB Cell Bank (Tokyo, Japan), the human hepatic cancer cell line HepG2 (RRID: CVCL_0027) was obtained from ATCC (Manassas, VA, USA), and the human embryonic kidney fibroblast line HEK293T (RRID: CVCL_0063) was also obtained from ATCC. Cells were cultivated in a cell incubator at 37°C with 5% CO_2_ at 95% humidity with DMEM that supplemented with 10% (v/v) fetal bovine serum (FBS) (Thermo Fisher Scientific, Waltham, MA, USA), MEM nonessential amino acids solution (Thermo Fisher Scientific), and penicillin-streptomycin (Thermo Fisher Scientific), which were passaged for an average of 3 days. Cells were authenticated by STR profiling at the time of receipt and periodically thereafter and tested routinely for mycoplasma contamination before use. Cell transfection was conducted using Lipofectamine 2000 (Thermo Fisher Scientific) in Opti-MEM medium (Thermo Fisher Scientific). Cell numbers at different timepoints were determined using a blood-counting chamber. D-serine (Sangon Biotech, Shanghai, China) and L-glutamine (Sangon Biotech) were used to determine the influence of SLC1A4 on amino acid influx and mTOR signaling.

### 2.4. Plasmid Construction

Total RNA of Huh7 cells was extracted using TRIzol reagent (Thermo Fisher Scientific), which was proceeded with reverse transcription PCR. The open reading frame of the AKT gene was amplified using the high-fidelity PCR kit KOD-Plus-Neo (Toyobo, Tokyo, Japan), which was inserted into p3 × Flag-CMV vector (expressing Flag-tag). Plasmids were amplified in *Escherichia coli* DH5*α* and sequenced at Sangon Biotech. Specific primers used for constructing Flag-AKT plasmid are listed below: GGGGTACC ATG AGC GAC GTG GCT ATT GTG AAG (forward primer) and GCTCTAGA GGC CGT GCC GCT GGC CGA GTA GGAG (reverse primer). Gene silencing plasmids were constructed using pGreenPuro shRNA Cloning and Expression Lentivector (SBI, Palo Alto, CA, USA). The targeted sequence for silencing SLC1A4 is GAA TTG TTC TGC CAC TTAT, and the sequence of negative control is ACT ACC GTT GTT ATA GGTG [22]. Moreover, pGL-c-Myc, pGL-EpCAM, and pRL-CMV plasmids were obtained from Addgene (Watertown, MA, USA).

### 2.5. Lentiviral Package and Infection

To stably silence SLC1A4 expression, lentiviruses were packaged and used for infecting hepatic cancer cells [23]. In brief, HEK293T cells in the logarithmic phase were seeded into 100 mm cell culture dishes to ensure the cell coverage at 60%–70%. After cultivation for 24 h, 8.0 µg of gene silencing plasmid, 8.0 µg of lentiviral packaging plasmid psPAX2, and 2.7 µg of envelope plasmid pMD2.G were mixed into 300 µL of Opti-MEM medium, and 18 µL of Lipofectamine 2000 was mixed into 300 µL of Opti-MEM medium. After incubation at room temperature for 5 min, the plasmid mixture and Lipofectamine 2000 mixture were mixed together and incubated at room temperature for 20 min. Then, the mixture was added to the medium of cultured HEK293T cells. After transfection for 48 h, supernatants of cell culture containing lentivirus were collected, which were filtered using 0.45 µm filters. The purified lentivirus was added to the culture medium of hepatic cancer cells. After infection with lentivirus for 72 h, cells were continuously treated with puromycin (Thermo Fisher Scientific) at 5 µg/mL to screen the stably infected cells.

### 2.6. Quantitative Real-time PCR (qRT-PCR)

Total RNA was extracted using TRIzol reagent. Reverse transcription PCR was conducted using PrimeScript RT reagent kit gDNA Eraser (Perfect Real Time) (Takara, Kusatsu, Japan), and qRT-PCR was conducted using SYBR Premix Ex Taq II (Tli RNaseH Plus) kit (Takara). GAPDH was used as the internal control, and the data of qRT-PCR was analyzed by the 2^(−*ΔΔ*Ct)^ method. The specific primers used for qRT-PCR are listed in Table 1.

### 2.7. Western blot

Tissue samples or cultured cells were rinsed using ice-cold PBS, which were lysed using RIPA buffer (Thermo Fisher Scientific) supplemented with phosphatase inhibitor (Thermo Fisher Scientific) and protease inhibitor cocktail (Thermo Fisher Scientific). After incubation on ice for 30 min, cell lysates were centrifuged at 16,000 × *g* and 4°C for 15 min, which were proceeded for protein concentration by the BCA method. Then, total protein were run on SDS–PAGE gels (10%), transferred onto PVDF membranes (Merck Millipore, Darmstadt, Germany), blocked with 5% skim milk (Sigma-Aldrich, St. Louis, MO, USA), and sequentially incubated with the primary antibodies at 4°C overnight and the secondary antibodies at room temperature for 2 h. Then, protein bands on PVDF membranes were examined using the SuperSignal West Pico Chemiluminescent Substrate kit (Thermo Fisher Scientific). Following antibodies were used for conducting Western blot: rabbit anti-SLC1A4 (8442S, CST, Danvers, MA, USA), mouse anti-GAPDH (clone 6C5, MAB374, Merck Millipore), mouse anti-Flag (clone M2, F3165, Sigma-Aldrich), rabbit anti-c-Myc (clone E5Q6W, 18583S, CST), rabbit anti-EpCAM (clone E6V8Y, 93790S, CST), rabbit anti-*β*-catenin (clone D10A8, 8480S, CST), rabbit anti-p-AKT (4060S, CST), rabbit anti-AKT (4691S, CST), mouse anti-p-mTOR (clone 59, sc-293133, Santa Cruz Technology, Dallas, TX, USA), mouse anti-mTOR (clone 30, sc-517464, Santa Cruz Technology), goat anti-mouse IgG (HRP-linked) (AP124P, Merck Millipore), and goat anti-rabbit IgG (HRP-linked) (AP132P, Merck Millipore).

### 2.8. Co-Immunoprecipitation (Co-IP)

Co-IP was conducted to examine the ubiquitin level of AKT according to the reported protocol [24]. In brief, cells were cultivated with 25 μM MG132 (APExBIO, Houston, TX, USA) for 6 h before protein collection, which was lysed with Pierce IP Lysis Buffer (Thermo Fisher Scientific) supplemented with a proteinase inhibitor cocktail. Supernatants of protein lysis were obtained by centrifuging at 16,000 × *g* for 15 min at 4°C. Then protein supernatants were diluted to 2 μg/μL with ice-cold PBS, immunoprecipitated with the rabbit anti-AKT antibody (4691S, CST) at 4°C overnight, and incubated with Protein G Agarose beads (Merck Millipore) at 4°C for 2 h. The precipitated beads were washed with ice-cold PBS and boiled in 2 × Laemmli Sample Buffer (Bio-Rad, Hercules, CA, USA) for 5 min, and the supernatants were collected and proceeded with Western blot.

### 2.9. Dual-Luciferase Reporter Assay

After cell seeding in a 12-well plate for 24 h, 300 ng of the promoter plasmid pGL-c-Myc or pGL-EpCAM, 20 ng of control plasmid pRL-CMV, and 700 ng of gene expression plasmids were mixed in 50 µL of Opti-MEM medium. Meanwhile, 3 µL of Lipofectamine 2000 was mixed into 50 µL of Opti-MEM medium at room temperature for 5 min. Then, the plasmid mixture and Lipofectamine 2000 mixture were mixed together and incubated at room temperature for 20 min, which were proceeded with cell transfection for 36 h. The transcriptional activity of promoters was examined using a Dual-Luciferase Reporter 1000 Assay System kit (Promega, Madison, WI, USA).

### 2.10. Measurement of D-Serine

An average of 3.0 × 10^5^ cells in the logarithmic phase were seeded into per well of a 6-well plate using DMEM medium without the supplement of MEM nonessential amino acids solution. About 48 h later, D-serine solution at a final concentration of 50 μM was added into the culture medium, and cells were further cultivated in a cell incubator for 60 min. Then, concentrations of intracellular D-serine were examined using a DL-Serine Assay Kit (Abcam, Cambridge, UK) according to the following protocol: (1) Preparation of standards: prepare a 200 µM solution of D-serine, add 0, 2, 4, 6, 8, and 10 µL of the 200 µM working solution into wells to generate 0, 400, 800, 1200, 1600, and 2000 pmol of D-Serine, and adjust the volume of per well to 60 µL using Assay Buffer LII/Serine Assay Buffer. (2) Preparation of samples: homogenized the cultured cells (about 1 × 10^6^) as soon as possible using 100 µL of ice-cold Assay Buffer LII/Serine Assay Buffer and centrifuged cell lysates at 15,000 × *g* for 10 min at 4°C to obtain the supernatants. Supernatants were further pretreated with Sample Cleanup Mix at a 1:25 ratio with an incubation at 37°C for 15 min, which were filtered using 10 kDa MWCO Spin Columns and further centrifuged at 10,000 × *g* for 10 min. (3) Added 10 µL of pretreated, filtered samples to per well of a black and flat bottom 96-well plate and adjusted the volume to 60 µL/well with Assay Buffer LII/Serine Assay Buffer. (4) Preincubated the 96-well plate at 37°C for 10 min and added 40 µL of the D-serine Only & Standards Reaction Mix to each well containing the standards or samples. (5) Incubated the plate at 37°C for 60 min in the dark and measured the fluorescence of samples and standards curve wells at *Ex*/*Em* = 535/587. (6) Analyzed the experimental data.

### 2.11. Transwell Experiments

Transwell chambers characterized with 8.0-μm PET membrane pores in 24-well plates (Corning Incorporated, Corning, NY, USA) were used in this study. After transfection with plasmids for 24 h, cells were starved with the serum-free DMEM medium for another 24 h, digested with trypsin, and resuspended with the serum-free DMEM medium. Then, 650 μL of DMEM medium supplemented with 10% (v/v) FBS was added to the lower chamber of Transwell, and 100,000 cells in 100 μL of serum-free DMEM medium were added to the upper chamber. After cultivation in a cell incubator with 5% CO_2_ at 37°C for 24 h, cells in Transwells were fixed with 4% paraformaldehyde at 4°C for 30 min and stained with 0.2% (m/v) crystal violet (Sigma-Aldrich) at room temperature for 15 min. The residue cells in the upper chambers were removed using medical cotton swabs, and the migrated cells in the lower chambers of Transwell were observed and captured under an IX73 inverted light microscope. The number of migrated cells was counted for statistical analysis.

### 2.12. Cultivation of Cancer Stem Spheres

After transfection with plasmids for 24 h, cells were digested with trypsin, rinsed with ice-cold PBS, and dispersed with the serum-free DMEM/F12 medium that supplemented with 20 ng/mL of EGF (Thermo Fisher Scientific), 20 ng/mL of FGF (Thermo Fisher Scientific), and 2% (v/v) B27 (Thermo Fisher Scientific). Then, 10,000 cancer cells in 2 mL of the serum-free medium were added to a 35-mm low-attachment cell culture plate (Corning Incorporated) and cultivated in a cell incubator with 5% CO_2_ at 37°C for 3 weeks. The serum-free medium for cultivating cancer stem spheres was refreshed every 3 days by centrifuging at 150 × *g* and room temperature for 3 min. Stem spheres formed by cancer cells were tracked and statistically analyzed based on the expression level of green fluorescence protein (GFP).

### 2.13. Immunofluorescence Staining

Rabbit anti-*β*-catenin (clone D10A8, 8480S) and Alexa Fluor 555 Phalloidin (8953S, CST) were used to assess *β*-catenin and F-actin levels, respectively. The simplified procedures were listed below: (1) An average of 1.5 × 10^5^ cells was seeded into a 35-mm confocal dish. (2) After cultivation for 48 h, cells were rinsed with ice-cold PBS and fixed with 4% paraformaldehyde at 4°C for 30 min. (3) Cells were incubated with 0.33 μM phalloidin at room temperature for 15 min or rabbit anti-*β*-catenin at room temperature for 90 min. Specifically, after incubation with rabbit anti-*β*-catenin, cells were further incubated with donkey anti-rabbit IgG (H + L) highly cross-adsorbed secondary antibody conjugated with Alexa Fluor 594 (A-21207, Thermo Fisher Scientific) at room temperature for 90 min. (4) Before staining the cell nucleus with DAPI in the dark at room temperature for 5 min, cells in confocal dishes were rinsed with PBS for 5 min. (5) The staining images of cells were observed and captured using a TI-E + A1 SI confocal microscope (Nikon, Tokyo, Japan).

### 2.14. Animal Experiments

The 6-week-old NCG mice (Strain NO. T001475, GemPharmatech, Nanjing, China) were fed under standard conditions in a pathogen-free environment at the Animal Care Facility of the Second Xiangya Hospital. Mice were randomly divided into two groups, with three mice in each group, and housed in a condition with a 12-h light/dark cycle. In total, 1,000,000 cells in 100 μL of the sterile PBS were administrated into NCG mice via the tail vein injection, and fluorescence in live mice was detected and traced using the IVIS Lumina XR live animal imager (PerkinElmer Waltham, MA, USA). Mice were sacrificed by cervical dislocation after cell injection for 30 days when they experienced a sharp decrease in activity, water, and diet intake. Protocols used in animal experiments were approved by the Animal Care and Experiment Committee of the Second Xiangya Hospital. All applicable international, national, and/or institutional guidelines for the care and use of animals were followed.

### 2.15. Statistical Analysis

GraphPad Prism 8 (GraphPad Software, La Jolla, CA, USA) was used to conduct statistical analyses and generate statistical graphs. Student's two-sided *t*-test was used to compare the significance between two groups, and one-way ANOVA was used to determine differences among multiple groups. Kaplan–Meier statistical method was applied to generate the 5-year overall survival curves. Experiments were repeated two to three times with similar results, and data are presented as mean ± SD with at least three biological replicates. Significant differences among groups were considered at *⁣*^*∗*^*p* < 0.05, *⁣*^*∗∗*^*p* < 0.01, *⁣*^*∗∗∗*^*p* < 0.001, and not-significant (ns).

## 3. Results

### 3.1. High Expression of SLC1A4 Predicts Poor Prognosis of HCC

**Figure 1:**
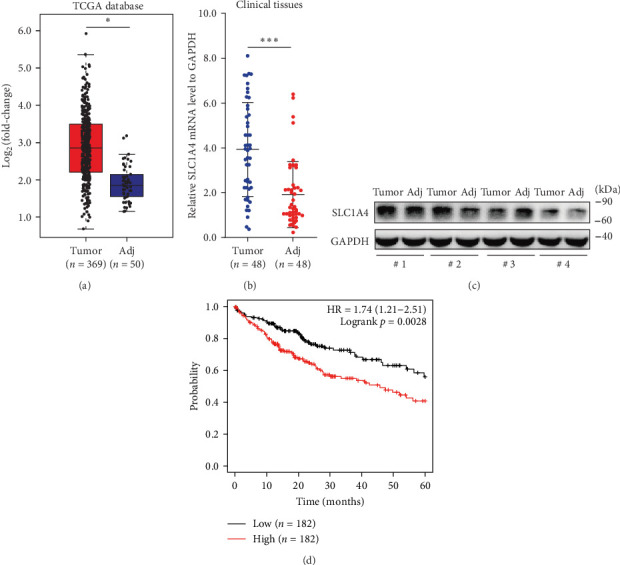
Expression profile and influence on prognosis of SLC1A4 in HCC. (a) Scatter plots showing the expression difference of SLC1A4 transcripts between tumor tissues (*n* = 369) and adjacent tissues (*n* = 50) of HCC in the TCGA database. (b) Scatterplot showing the expression difference of SLC1A4 mRNA between tumor tissues (*n* = 48) and adjacent tissues (*n* = 48) of clinical HCC samples. (c) Protein level of SLC1A4 in tumor tissues and adjacent tissues derived from 4 representative HCC patients, as assayed by Western blot. (d) Kaplan–Meier analysis for the 5-year overall survival of HCC presenting high (*n* = 182) and low (*n* = 182) levels of PIK3CB transcripts in the TCGA database, as analyzed using the log-rank test. High and low expression groups of SLC1A4 were determined according to the median value. Data were analyzed by Student's two-sided *t*-test and are presented as mean ± SD (a and b). *⁣*^*∗*^*p* < 0.05 and *⁣*^*∗∗∗*^*p* < 0.001. HCC, hepatocellular carcinoma.

The results of integrative bioinformatics and functional profiling indicated that SLC1A4 may involve in regulating cancer phenotypes, immune regulation, and drug resistance in HCC [19], but the regulatory role and mechanism of SLC1A4 in HCC is still obscure. Through analyzing HCC data in the TCGA database, we revealed that the transcript level of SLC1A4 in tumor tissues of HCC was significantly higher than that in adjacent tissues (Figure 1a). Through conducting qRT-PCR, we verified the high expression of SLC1A4 in tumor tissues of clinical HCC samples (Figure 1b). Meanwhile, the protein level of SLC1A4 in tumor tissues of HCC examined by Western blot was also uncovered to be generally higher than that in adjacent tissues (Figure 1c). These findings identified the high expression of SLC1A4 in HCC, indicating that SLC1A4 may promote the development and progression of HCC. We further evaluated the influence of SLC1A4 on 5-year overall survival of HCC. It turned out that a high level of SLC1A4 might predict the poor prognosis of HCC (Figure 1d). All these experimental data suggested the oncogenic role and regulation of SLC1A4 in HCC.

### 3.2. SLC1A4 Promotes the Expression of c-Myc and EpCAM Through Activating AKT Signaling

**Figure 2:**
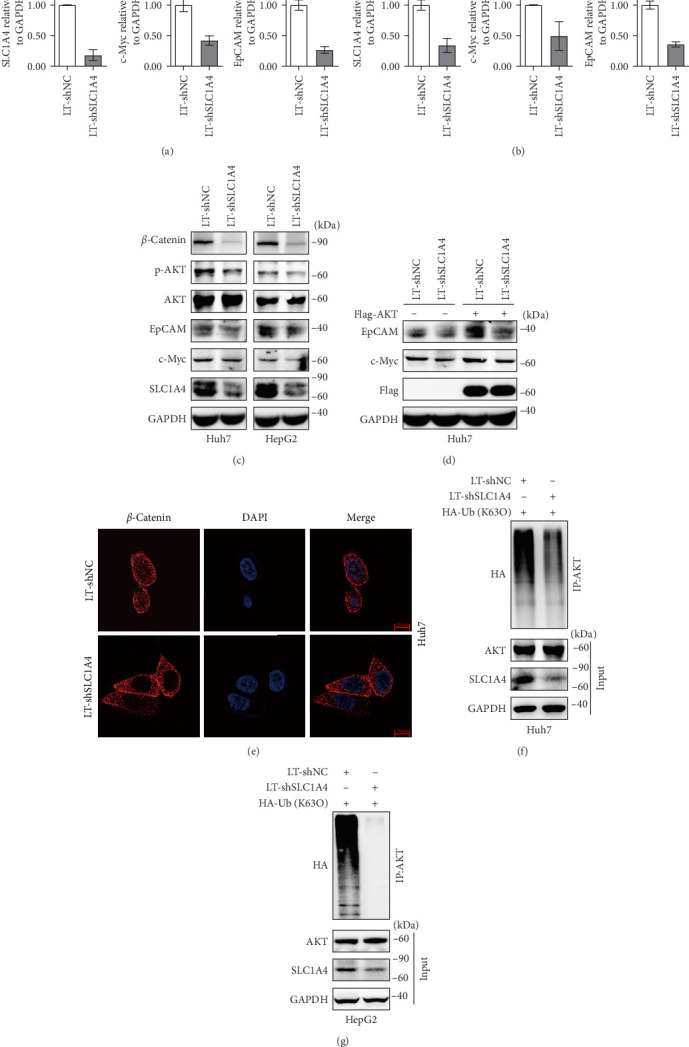
Regulatory mechanism of SLC1A4 on AKT signaling. (a and b) qRT-PCR for mRNA levels of SLC1A4, c-Myc, and EpCAM in Huh7 and HepG2 cells. Cells were stably infected with lentivirus for silencing SLC1A4 expression. (c) Western blot for the levels of *β*-catenin, p-AKT, AKT, EpCAM, c-Myc, and SLC1A4 in Huh7 and HepG2 cells. Cells were stably infected with lentivirus for silencing SLC1A4 expression. (d) Western blot for EpCAM, c-Myc, and Flag levels in Huh7 cells. The stably infected Huh7 cells with lentivirus for silencing SLC1A4 expression were further transfected with Flag-AKT plasmid or control Flag-vector for 48 h. (e) Immunofluorescence staining for protein level of *β*-catenin in nuclei of Huh7 cells with SLC1A4 knockdown. Scale bar, 10 μm. (f and g) Co-IP for K63-dependent ubiquitin level of AKT protein in Huh7 (f) and HepG2 (g) cells with the knockdown of SLC1A4. The stably infected Huh7 cells with lentivirus for silencing SLC1A4 expression were further transfected with pHA-Ub (K63O) for 48 h, and cell lysates were immunoprecipitated with an anti-AKT antibody and examined for HA levels. Data were analyzed by Student's two-sided *t*-test and are presented as mean ± SD with three biological replicates. *⁣*^*∗*^*p* < 0.05, *⁣*^*∗∗*^*p* < 0.01, and *⁣*^*∗∗∗*^*p* < 0.001.

To uncover the regulatory mechanism of SLC1A4 in HCC, gene silencing plasmid LT-shSLC1A4 and the negative control LT-shNC were constructed and administrated into hepatic cancer cells by lentiviral infection. The results of qRT-PCR and Western blot revealed the profound effect of LT-shSLC1A4 plasmid on silencing SLC1A4 expression in Huh7 and HepG2 cells (Figure 2a–c). AKT and *β*-catenin regulate malignant transformation and prognosis of multiple cancers. Specifically, the PTEN-AKT signaling pathway may control the activation of stem cells and cancer progression by promoting nuclear localization of *β*-catenin [25, 26]. SLCs may regulate cancer phenotypes represented by proliferation, invasion, and metastasis through regulating the PI3K/AKT signaling pathway [27–29]. However, whether SLC1A4 may regulate the development and progression of HCC through regulating signal transduction mediated by AKT and *β*-catenin is still unclear. Through conducting Western blot, we found that the levels of *β*-catenin and p-AKT, as well as the downstream factors, including c-Myc and EpCAM, were decreased with the knockdown of SLC1A4 in Huh7 and HepG2 cells (Figure 2c), indicating the potential mechanism regulated by SLC1A4 in HCC. Through conducting qRT-PCR, we also observed the decreased mRNA levels of c-Myc and EpCAM in SLC1A4-silenced Huh7 and HepG2 cells (Figure 2a,b). Notably, the introduction of exogenous AKT increased the protein levels of c-Myc and EpCAM in SLC1A4-silenced Huh7 cells (Figure 2d). Moreover, the *β*-catenin protein in the nuclei of Huh7 cells was almost disappeared with the knockdown of SLC1A4 (Figure 2e), suggesting for the inactivation of *β*-catenin in hepatic cancer cells. Lysine 63 (K63)-dependent ubiquitination leads to phosphorylation activation of AKT protein [30, 31]. Through conducting co-IP experiments, we uncovered that the K63-dependent ubiquitin level of AKT was decreased with the knockdown of SLC1A4 in Huh7 cells (Figure 2f,g). All experimental data suggested the positive role of SLC1A4 in activating AKT signaling in hepatic cancer cells.

**Figure 3:**
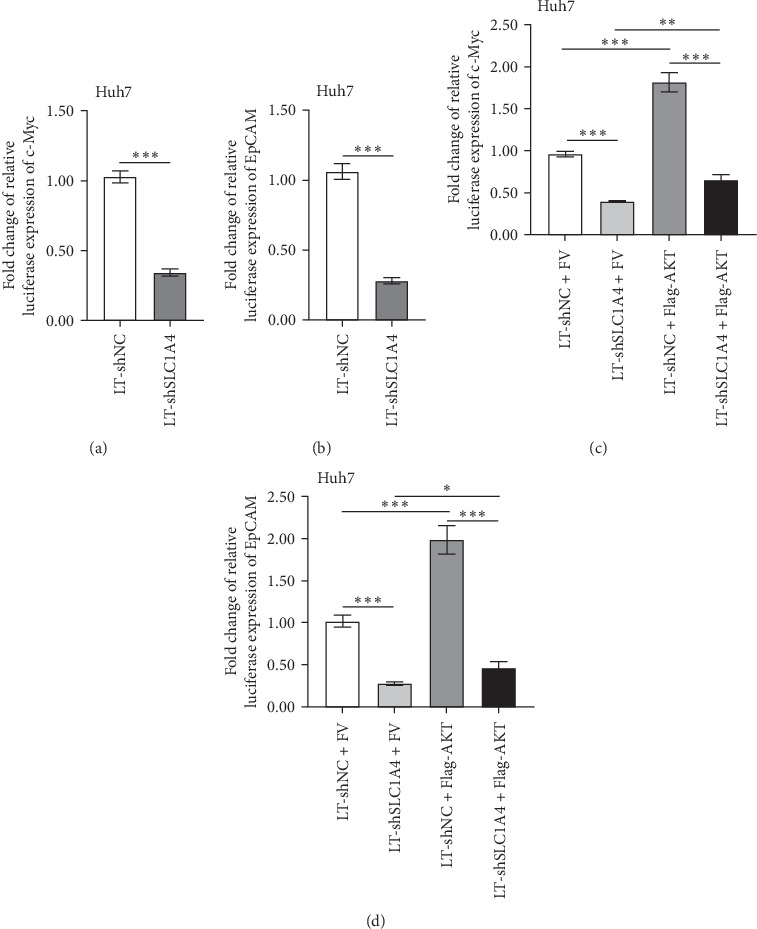
The regulatory effect of SLC1A4 and AKT on transcriptional activity of c-Myc and EpCAM promoters. (a and b) Fold change of luciferase expression of pGL-c-Myc (a) and pGL-EpCAM (b) plasmids in Huh7 cells with the knockdown of SLC1A4. (c and d) Fold change of luciferase expression of pGL-c-Myc (c) and pGL-EpCAM (d) plasmids in Huh7 cells. The stably infected Huh7 cells with lentivirus for silencing SLC1A4 expression were further transfected with Flag-AKT plasmid for 36 h. Data were analyzed by Student's two-sided *t*-test or one-way ANOVA and are presented as mean ± SD with three biological replicates. *⁣*^*∗*^*p* < 0.05, *⁣*^*∗∗*^*p* < 0.01, and *⁣*^*∗∗∗*^*p* < 0.001.

To approve our findings, the transcriptional activity of c-Myc and EpCAM promoters in hepatic cancer cells with different levels of SLC1A4 was examined by conducting dual-luciferase reporter assays. It turned out that silencing SLC1A4 significantly decreased the transcriptional activity of c-Myc and EpCAM promoters in Huh7 cells (Figure 3a,b). Moreover, the downregulated transcriptional activity of c-Myc and EpCAM promoters in SLC1A4-silenced Huh7 cells was upregulated again by the transfection of exogenous AKT (Figure 3c,d). These data approved the regulatory mechanism that SLC1A4 activates AKT signaling in HCC.

### 3.3. SLC1A4 Promotes Amino Acid Influx and mTOR Activation in Hepatic Cancer Cells

**Figure 4:**
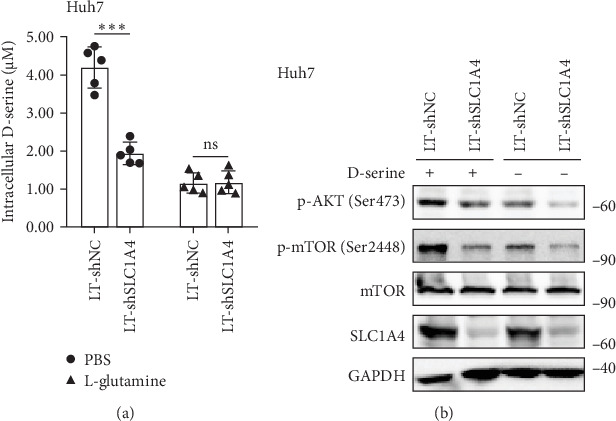
Influence of SLC1A4 on D-serine uptake and mTOR activation in hepatic cancer cells. (a) Uptake of D-serine into Huh7 cells with different levels of SLC1A4 was measured over a period of 60 min. D-serine at a final concentration of 50 μM and L-glutamine at a final concentration of 100 μM were used to incubate with Huh7 cells. (b) Western blot for p-AKT, p-mTOR, mTOR, and SLC1A4 levels in Huh7 cell lysates. The stably infected cells with lentivirus were cultivated in a medium with or without 50 μM D-serine for 24 h. Data were analyzed by Student's two-sided *t*-test are presented as mean ± SD with five biological replicates. *⁣*^*∗∗∗*^*p* < 0.001.

As a physiologic regulator of D-serine metabolism, SLC1A4 is a major D-serine uptake system in astrocytes [32, 33]. Interestingly, the uptake of D-serine sustains mTOR activity and cell proliferation [34, 35]. To further uncover the oncogenic mechanism of SLC1A4, we examined amino acid influx and mTOR activity in Huh7 cells. It turned out that the intracellular level of D-serine was decreased with the knockdown of SLC1A4 (Figure 4a). The administration of L-glutamine inhibits the absorption of D-serine [36], which was verified and used as a control in our study (Figure 4a). Notably, p-mTOR and p-AKT levels in Huh7 cells were decreased with the downregulation of SLC1A4 and deprivation of D-serine (Figure 4b). Thus, amino acid metabolism represented by D-serine involves in AKT activation and malignant transformation of HCC.

### 3.4. SLC1A4 Promotes Cancer Phenotypes of Hepatic Cancer Cells

**Figure 5:**
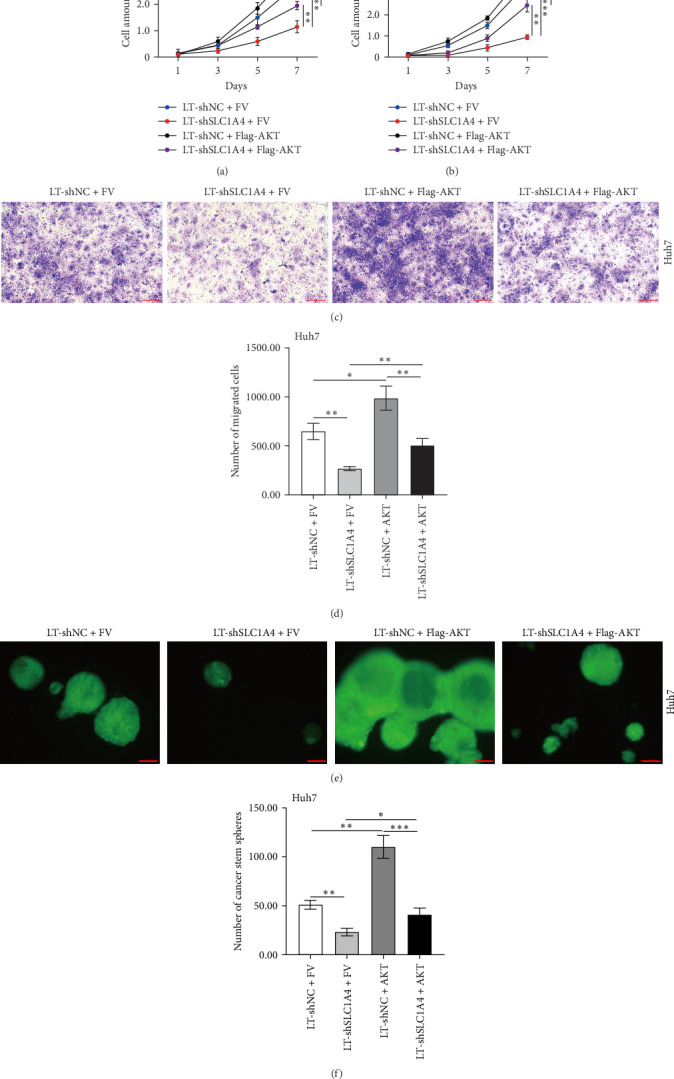
Regulatory effect of SLC1A4 on phenotypes of hepatic cancer cells. (a and b) Proliferation of Huh7 (a) and HepG2 (b) cells with the knockdown of SLC1A4. (c and d) Images (c) and statistical analysis (d) of migrated Huh7 cells with the knockdown of SLC1A4. Scale bar, 100 μm. (e and f) Images (e) and statistical analysis (f) of cancer stem spheres formed by Huh7 cells with the knockdown of SLC1A4. Scale bar, 1 mm. Data were analyzed by one-way ANOVA and are presented as mean ± SD with three biological replicates. *⁣*^*∗*^*p* < 0.05, *⁣*^*∗∗*^*p* < 0.01, and *⁣*^*∗∗∗*^*p* < 0.001.

Next, we explored the regulatory effect of SLC1A4 and AKT on cancer phenotypes of hepatic cancer cells. It turned out that silencing SLC1A4 expression significantly inhibited the proliferation of Huh7 and HepG2 cells (Figure 5a,b). However, the suppressed cell proliferation caused by the knockdown of SLC1A4 was successfully reversed by the forced expression of AKT (Figure 5a,b). Meanwhile, the migratory ability of Huh7 cells in Transwells was inhibited by the knockdown of SLC1A4, which was also reversed by the transfection of the Flag-AKT plasmid (Figure 5c,d). Therefore, SLC1A4 may promote the proliferation and migration of hepatic cancer cells through activating AKT signaling. Moreover, the number and size of cancer stem cell spheres formed by Huh7 cells were decreased with the knockdown of SLC1A4 (Figure 5e,f). Thus, SLC1A4 enhances the cancer stemness of hepatic cancer cells. Interestingly, the decreased cancer stemness triggered by the downregulation of SLC1A4 was enhanced and reinforced by the introduction of exogenous AKT again (Figure 5e,f). These data not only revealed the oncogenic role of SLC1A4 in HCC but also underlined that the activation of AKT signaling is an important mechanism for SLC1A4 to exert the oncogenic role in HCC.

### 3.5. SLC1A4 Promotes Malignant Transformation of HCC

**Figure 6:**
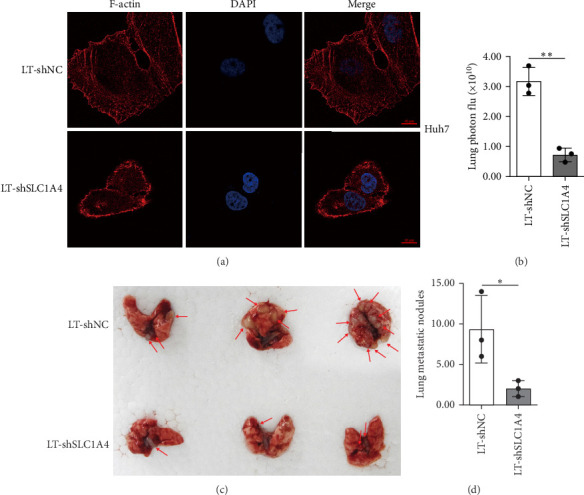
Influence of SLC1A4 on cytoskeletal remodeling and cancer metastasis. (a) Immunofluorescence staining for F-actin using phalloidin in Huh7 cells with SLC1A4 knockdown. Nuclei were stained with DAPI. Scale bar, 10 μm. (b) Statistical analysis of GFP fluorescence in NCG mice on 30 days post tail vein injection of Huh7 cells. Huh7 cells were stably infected with lentivirus for silencing SLC1A4 expression. (c and d) Image (c) and statistical analysis (d) of formed lung metastatic nodules in NCG mice on 30 days post tail vein injection of Huh7 cells. The red arrows in (c) present the lung metastatic nodules. Huh7 cells were stably infected with lentivirus for silencing SLC1A4 expression. Data were analyzed by Student's two-sided *t*-test and are presented as mean ± SD with three biological replicates. *⁣*^*∗*^*p* < 0.05 and *⁣*^*∗∗*^*p* < 0.01.

Epithelial–Mesenchymal Transition (EMT) and metastasis are two critical indexes for evaluating the malignant transformation of malignances. Notably, cytoskeletal reorganization is an important characteristic of cancer EMT [22]. To examine the effect of SLC1A4 on regulating the EMT of hepatic cancer cells, immunofluorescence staining of F-actin using Alexa Fluor 555 Phalloidin was conducted in this study. Compared with that in control cells, the decreased lamellipodia and stress fibers were observed in SLC1A4-silenced Huh7 cells (Figure 6a). Meanwhile, the protein levels of *β*-catenin and EpCAM in Huh7 and HepG2 cells were decreased with the knockdown of SLC1A4 (Figure 2c), approving the promoting role of SLC1A4 in EMT of hepatic cancer cells [37–39]. Therefore, SLC1A4 may promote the EMT of hepatic cancer cells. The regulatory effect of SLC1A4 on the metastasis potential of hepatic cancer cells was evaluated by conducting tail vein injection. It turned out that silencing SLC1A4 significantly decreased the fluorescence intensity of lentivirus-infected Huh7 in the lung regions of NCG mice (Figure 6b). Meanwhile, the number of formed lung metastatic nodules in the control group was significantly more than that in the SLC1A4-silenced group (Figure 6c,d). These data suggested that SLC1A4 may promote metastasis of HCC.

## 4. Discussion

HCC greatly damages the health status and life quality of human beings, which may be caused by hepatitis virus, alcoholism, aflatoxin, and so on [40]. The difficulty in early diagnosis and the shortage of therapeutic methods for advanced disease result in a high mortality rate of HCC. SLCs are a group of membrane transport proteins with over 400 members, mediating the transportations of multiple substrates across biological membranes [41, 42]. SLC transporters are widely expressed in human organs, such as the liver, intestine, kidney, and even brain, testes, and placenta. Through regulating the delivery of substrates, SLC transporters maintain cellular homeostasis and influence cellular uptake of nutrients and drugs [43, 44]. Some drugs targeting SLC transporters have been developed for the associated diseases [45, 46]. In this study, an SLC transporter named SLC1A4 was found to be highly expressed in tumor tissues of HCC, and a high level of SLC1A4 was tightly associated with the poor prognosis of HCC. These data present the regulatory potential of SLC1A4 in the development and progression of HCC.

The activation of the AKT signaling pathway is closely associated with cancer malignancy, drug resistance, immune evasion, and multiple biological characteristics and clinical phenomena of multiple cancers [47–50]. Meanwhile, the aberrations in the Wnt/*β*-catenin signaling pathway are generally linked with cancer phenotypes, including proliferation, differentiation, migration, and cancer stemness, exerting crucial roles in regulating tumorigenesis and cancer therapy response [51, 52]. In addition to being an important marker of cancer stemness, *β*-catenin is found to be a transcription factor for oncogenes represented by Cyclin D1, c-Myc, and PD-L1 [22, 53, 54]. Interestingly, EpCAM has been identified to be a hepatic stem cell marker that is regulated by both AKT and Wnt/*β*-catenin signaling in HCC [55, 56]. In this study, the levels of p-AKT, *β*-catenin, c-Myc, and EpCAM in hepatic cancer cells were decreased with the knockdown of SLC1A4, and the K63-dependent ubiquitin level of AKT protein was decreased with the knockdown of SLC1A4. Notably, transcriptional levels of c-Myc and EpCAM promoters in hepatic cancer cells were downregulated by the decreased level of SLC1A4, which was reversed by the transfection of the Flag-AKT plasmid. Moreover, cancer phenotypes of hepatic cancer cells, including proliferation, migration, and cancer stemness, were suppressed by the downregulation of SLC1A4, which was reversed by the introduction of exogenous AKT. Ultimately, silencing SLC1A4 decreased the expression of lamellipodia and stress fibers in hepatic cancer cells and the metastasis potential of hepatic cancer cells. Thus, SLC1A4 may enhance c-Myc and EpCAM expression by promoting signal transduction mediated by AKT and *β*-catenin, thus enhance the malignant transformation of HCC. These findings reveal the regulatory mechanism of SLC1A4 in promoting the development and progression of HCC. Actually, other SLCs, such as SLC38A1 and SLC12A8, are also involved in cancer development and drug resistance by regulating the AKT signaling [57, 58]. It seems that some SLCs may possess some specific structures that can target and regulate the expression and stability of AKT, which need to be further studied.

Deregulated anabolism/catabolism of amino acids, especially glutamine, serine, and glycine, are metabolic regulators in supporting the survival and proliferation of cancer cells [59, 60]. Interestingly, mTOR signaling promotes the absorption of amino acids, which in turn drives the activation of mTORC1 [35, 61]. The regulation of some SLCs, such as SLC7A11, SLC1A5, and SLC38A9, are involved in cancer progression [35, 62]. In this study, D-serine uptake was decreased with the knockdown of SLC1A4 in hepatic cancer cells. Moreover, p-AKT and p-mTOR levels in hepatic cancer cells were lowered by the downregulation of SLC1A4 and deprivation of D-serine. Thus, the malignant transformation of HCC may be tightly associated with the metabolism of amino acids, especially for that of nonessential amino acids.

The innovations and main findings of this study are imbedded in the following points: First, SLC1A4 was found to promote the malignant transformation of HCC. Second, AKT signaling and amino acid inflex represented by D-serine were uncovered to be regulated by SLC1A4, presenting the regulatory mechanism of SLC1A4 on promoting malignant transformation of HCC. Third, the transcriptional activity of c-Myc and EpCAM promoters was identified to be promoted by SLC1A4. The findings in this study may provide new sights for improving the diagnosis of early HCC and the therapeutic efficacy of advanced HCC. However, whether AKT or *β*-catenin may function as transcription factors of c-Myc and EpCAM regulated by SLC1A4 is still unclear, and the potential regulation of SLC1A4 in tumor microenvironment represented by cancer immunity is not referred to in this study.

## Figures and Tables

**Table 1:** Specific primers used for conducting qRT-PCR.

Genes	Primers (5′-3′)	Amplicon (bp)
SLC1A4	Forward: GCCTACTTTGGCCTCACCAC	221
	Reverse: GCATACGTACGGAAAGCTGC	

c-Myc	Forward: TACAACACCCGAGCAAGGAC	189
	Reverse: AGCTAACGTTGAGGGGCATC	

EpCAM	Forward: TGCTGGAATTGTTGTGCTGG	190
	Reverse: AAGATGTCTTCGTCCCACGC	

GAPDH	Forward: CCGCATCTTCTTTTGCGTCG	225
	Reverse: CGGTGCCATGGAATTTGCC	

Abbreviation: qRT-PCR, quantitative real-time PCR.

## Data Availability

All data related to the findings in this study are available upon reasonable request from the corresponding author.
